# Heat-related illness risk and associated personal and environmental factors of construction workers during work in summer

**DOI:** 10.1038/s41598-020-79876-w

**Published:** 2021-01-13

**Authors:** Takeyasu Kakamu, Shota Endo, Tomoo Hidaka, Yusuke Masuishi, Hideaki Kasuga, Tetsuhito Fukushima

**Affiliations:** Department of Hygiene and Preventive Medicine, Fukushima Medical University, Fukushima City, 1 Hikarigaoka, Fukushima, 960-1295 Japan

**Keywords:** Occupational health, Environmental health, Epidemiology

## Abstract

Heat-related illness (HRI) is a common occupational injury, especially in construction workers. To explore the factors related to HRI risk in construction workers under hot outdoor working conditions, we surveyed vital and environmental data of construction workers in the summer season. Sixty-one workers joined the study and the total number of days when their vital data during working hours and environmental data were recorded was 1165. Heart rate with high-risk HRI was determined using the following formula: 180 − 0.65 × age. As a result of the logistic regression analysis, age, working area, maximum skin temperature, and heart rate immediately after warming up were significantly positively related, and experience of construction was significantly negatively related to heart rate with high-risk HRI. Heart rate immediately after warming up may indicate morning fatigue due to reasons such as insufficient sleep, too much alcohol intake the night before, and sickness. Asking morning conditions may lead to the prevention of HRI. For occupational risk management, monitoring of environmental and personal conditions is required.

## Introduction

Amid the effects of global warming, heat-related health risks are increasing^1,2^. Deaths due to heat-related illness (HRI) have occurred at a rate of about 500 per year since 2010 when unusually hot summer affected Japan^3,4^. In the workplace, the impacts of heat exposure can be particularly harsh on outdoor workers, such as those in the agriculture, construction, mining and manufacturing industries^5^. In Japan, occupational HRI occurs in about 500 people every year, among whom 20 die^6^. The World Health Organization asserts that “any decline in a worker’s performance of daily activities due to heat, cold, or extreme weather should be considered a ‘health effect’ of climate conditions”^7,8^. Similarly, the ILO positions it as one of the major occupational accidents that should prevent HRI^9^. For example, in 2015, the Occupational Safety and Health Administration in the Unites States (U.S. OSHA) declared that workplace heat illness and death incidents may increase unless employers initiate comprehensive preventive programs^10^. The Japanese Ministry of Health, Labour and Welfare determined that prevention of occupational heat stress is an important goal in the 13th Occupational Safety and Health Program^11^. In addition, US. OSHA determined measures to prevent occupational HRI, such as monitoring heat conditions, workers’ conditions, adjustments of schedules and workload, scheduled breaks, and similar guidelines have been issued in Japan^12,13^.

Construction is the highest risk industry in terms of occupational HRI^14,15^. According to the National Institute for Occupational Safety and Health in the US (US. NIOSH), the highest number of HRI-related deaths has been reported in the construction industry for a long time^16^. In addition, the Japanese Ministry of Health, Labour and Welfare reported that about 40% of HRIs happened in the construction industry in 2018^6^. Several factors increase the risk of HRI. These include the constant use of machinery and powered tools, working on elevated surfaces, heavy workload, simple accommodation conditions near work sites, being temporarily employed by a subcontractor on a daily payment basis, and constant and direct exposure to sunlight^5^.

Wet-Bulb Globe Temperature (WBGT) is a validated empirical index of environmental heat stress and has ISO certifications (ISO 7933: 1984 and ISO 7243: 1989)^17–19^. Measuring WBGT in the workplace is a fundamental measure of HRI prevention^10,12^ and WBGT is used as a heat stress index in many studies^5,20^. On the other hand, HRI involves complex interactions between environmental heat strain, clothing, and human thermal physiology^21^. Recently, a wearable sensor for monitoring workers’ conditions has been developed^22^. US NIOSH recommends physiologic monitoring such as heart rate and/or core temperature of heat-exposed workers^16^. A wearable sensor enables workers to monitor their condition, making it possible to take a rest when they feel fatigue or to express that they feel unwell.

Continuous monitoring can make it possible to grasp the physical condition of all workers even from the remote place. Especially, its monitoring might be useful for construction workers because their working site is outdoor and they often work in small group or alone. To clarify HRI risk by using continuous monitoring data and personal background information is needed to lead effective management for construction workers. The aim of the present study was to explore the personal and environmental factors related to HRI risk using continuous monitoring data recorded by a wearable sensor in hot outdoor working conditions.

## Results

### Characteristics

The total number of person-time when vital data of the workers during working hours and environmental data were recorded was 1165. WBGT was measured for 42 days in Site 1 and 43 days in Site 2. On these 1165 person-time, a high heart rate indicative of HRI risk was observed on 102 person-time (8.7%).

We describe the workers’ personal data in Table 1 and the environmental data in Table 2. The mean age (standard deviation) was 48.4 (14.0) years, and median number of working days (25–75 percentile) was 14 (7–35). During the 85 days (Site 1 + Site 2), WBGT of over 31 °C was recorded on 49 days (57.6%) (21 days [50.0%] in Site 1 and 28 days [65.1%] in Site 2). When the personal data were compared between Site 1 and Site 2, only the proportion of hypertension (6 [17.1%] and 0 [0.0%]) showed significant difference. When the environmental data as median (25–75 percentile) (°C) were compared between Site 1 and Site 2, maximum WBGT (30.9 [28.7–32.3] and 32.6 [29.8–34.2], *p* = 0.004), minimum WBGT (25.7 [23.0–27.5] and 26.4 [25.1–28.7], *p* = 0.004), average WBGT (28.4 [25.5–30.1] and 30.2 [27.5–31.2], *p* = 0.014), maximum dry bulb temperature (33.7 [31.2–35.2] and 36.8 [32.5–39.4], *p* = 0.001), minimum dry bulb temperature (27.7 [24.9–29.3] and 27.9 [25.5–32.9], *p* = 0.042) and average dry bulb temperature (31.1 [28.0–32.7] and 33.0 [29.5–36.1], *p* = 0.003) were significantly higher in Site 2.

**Table 1:** Subject characteristics.

Item	Site 1	Site 2	*p* value
Number	35	26	
Age (year)	49.0 (13.2)	48.0 (15.1)	0.794
Total working days (days/person)	17 (5–32)	13 (5–35)	0.878
Duration of career in construction (year)	19 (8–28)	15 (9–26)	1.000
Hypertension	6 (17.1)	0 (0.0)	*0.033*
Diabetes	1 (2.9)	1 (3.8)	1.000
**Employer**			
Subcontractor	21 (60.0)	11 (42.3)	0.239
Sub and sub-sub-subcontractor	14 (40.0)	15 (57.7)	

Italic font describes significant difference (*p* < 0.05).

Mean (SD) or Median (25–75 percentile) or n (%).

*p* value was calculated by Student’s t-test, Wilcoxon rank sum test, chi-square test, or Fisher’s exact test.

**Table 2:** Environmental data.

Item	Site 1	Site 2	*p* value
Working days (days)	42	43	
**WBGT (°C)**			
Maximum	30.9 (28.7–32.3)	32.6 (29.8–34.2)	*0.004*
Minimum	25.7 (23.0–27.5)	26.4 (25.1–28.7)	*0.004*
Average	28.4 (25.5–30.1)	30.2 (27.5–31.2)	*0.014*
**Dry bulb temperature (°C)**			
Maximum	33.7 (31.2–35.2)	36.8 (32.5–39.4)	*0.001*
Minimum	27.7 (24.9–29.3)	27.9 (25.5–32.9)	*0.042*
Average	31.1 (28.0–32.7)	33.0 (29.5–36.1)	*0.003*
**Relative humidity (%)**			
Maximum	72.0 (67.9–79.0)	80.1 (63.6–99.9)	0.271
Minimum	50.2 (46.1–53.8)	49.2 (39.7–56.9)	0.568
Average	59.5 (54.7–64.9)	58.8 (48.4–73.2)	0.709

Italic font describes significant difference (*p* < 0.05).

n or median (25–75 percentile).

*P* value was calculated by using the Wilcoxon rank sum test.

The vital data of the 1165 days are shown in Table 3. When vital data as mean ± SD were compared between high and low risk groups, maximum heart rate (BPM) (165.9 ± 16.1 and 119.3 ± 16.0, *p* < 0.001), minimum heart rate (BPM) (58.1 ± 14.2 and 54.3 ± 6.4, *p* = 0.010) and heart rate immediately after warming up (BPM) (89.0 ± 23.2 and 81.8 ± 15.3, *p* = 0.004) were significantly higher in the high risk group. Skin temperature and energy consumption did not show a significant difference between the high and low risk groups. In a comparison of energy consumption among age groups, the age group of ≥ 60 years consumed significantly less energy (kcal), with a mean (SD) of 566.0 (176.4), compared to 744.9 (189.0), 771.0 (176.5), and 703 (184.5) in the age groups of < 40, 40–49, and 50–59 years, respectively.

**Table 3:** Vital data.

Item	High risk (n = 99)	Low risk (n = 1066)	*p* value
**Skin temperature (°C)**			
Maximum	37.0 ± 2.75	36.6 ± 1.63	0.097
Minimum	30.1 ± 2.50	30.4 ± 2.43	0.230
**Heart rate (BPM)**			
Maximum	165.9 ± 16.1	119.3 ± 16.0	< *0.001*
Minimum	58.1 ± 14.2	54.3 ± 6.40	*0.010*
After warming up	89.0 ± 23.2	81.8 ± 15.3	*0.004*
Energy consumption (kcal)	720.2 ± 216.3	692.1 ± 194.3	0.215

Italic font describes significant difference (*p* < 0.05).

Mean ± Standard deviation.

*p* value was calculated by using Student’s t test.

### Factors associated to heat-related illness risk

As a result of the logistic regression analysis, age (odds ratio [OR] 1.051, 95%CI 1.025–1.077), working in Site 2 (OR 2.485, 95%CI 1.410–4.381), maximum skin temperature (OR 1.217, 95%CI 1.074–1.378), and heart rate immediately after warming up (OR 1.031, 95%CI 1.018–1.045) were significantly positively related, and duration of career in construction (OR 0.977, 95%CI 0.954–0.999) was significantly negatively related, to heart rate with high-risk HRI (Table 4).

**Table 4:** Logistic regression of heat-related illness risk.

Item	Crude OR (95%CI)	Adjusted OR (95%CI)
Age	*1.023 (1.006–1.040)*	*1.051 (1.025–1.077)*
Working day	0.982 (0.963–1.001)	0.985 (0.964–1.006)
Duration of career in construction	0.999 (0.982–1.015)	*0.977 (0.954–0.999)*
Hypertension	1.211 (0.679–2.158)	1.166 (0.531–2.561)
Diabetes	1.037 (0.404–2.660)	1.384 (0.481–3.983)
Site 2	*2.214 (1.453–3.374)*	*2.485 (1.410–4.381)*
Sub and sub-subcontractors	*2.216 (1.464–3.354)*	1.554 (0.950–2.543)
Maximum skin temperature	*1.143 (1.027–1.272)*	*1.207 (1.065–1.367)*
Energy consumption	1.001 (0.999–1.002)	1.001 (0.999–1.002)
Heart rate after warming up	*1.024 (1.012–1.036)*	*1.033 (1.019–1.047)*
**Maximum WBGT (°C)**		
25–28	4.479 (0.556–36.098)	2.964 (0.355–24.784)
28–31	2.465 (0.321–18.932)	1.062 (0.131–8.640)
> 31	4.910 (0.666–36.208)	1.351 (0.165–11.088)
WBGT difference in a day	1.075 (0.936–1.234)	1.396 (0.17–11.452)

Italic font describes significant difference (*p* < 0.05).

*OR* odds ratio, *CI* confidence interval.

## Discussion

We investigated HRI risk in construction workers using their personal information, vital data and environmental data, and several factors were found to be risk factors of HRI. A previous study has recommended heat stress monitoring to prevent HRI^23^. Self-monitoring such as perceptual strain has been widely used to assess heat strain; however, it is said to do little to contribute to the protection of workers’ health^23^. Self-monitoring of workers’ conditions using their physiological responses has been determined to be most effective to prevent HRI^23^. Continuous objective monitoring is more effective for construction workers because their working site is outdoor and they often work in small group or alone. To use objective monitoring, early recognition of heat strain and management corresponds to the situation can be enabled even from the remote site. A wearable sensor for monitoring health conditions can assess physiological responses and its technology is continually developing^7,16,23^. Our result may indicate that combining data on environmental monitoring and those from wearable sensors during work contributes to the estimation of HRI risks, enabling better control of working load for HRI risk reduction.

Our previous study and other studies demonstrated that insufficient sleep is a risk factor of HRI^24–26^. In addition, insufficient sleep has been shown to be strongly related to fatigue^27^. Heart rate increase is correlated to fatigue during physical exercise^28^. Furthermore, fatigue due to insufficient sleep might cause acute increases in heart rate. Therefore, heart rate immediately after warming up may be a good index for HRI risk assessment. We used Japanese radio calisthenics for warming up. These are simple exercises that can be performed regardless of age and can move the muscles and joints effectively according to the rhythm of the radio. It is considered a therapeutic exercise to promote health in Japan. The required time for calisthenics is approximately 3 min, and it has an exercise intensity of 4–4.5 metabolic equivalents (METs)^29^. In the present study, however, all subjects wore vital sensors after warming up. Wearing sensors before warming up and monitoring heart rate while warming up would have been more effective for evaluating workers’ condition. After such evaluation, allocation of work could have been done according to workers’ physical condition.

Aging has been reported to be significantly related to increased HRI risk. On the other hand, our previous study indicated that younger workers are at a higher risk of HRI^24^. Generally, exertional HRI occurs in healthy young people, whereas classical HRI is more likely to occur in the elderly^30^. One possible reason is that younger people tend to pay little attention to preventive measures for HRI^31^. Another possible reason is that younger age groups tend to engage in more physically intensive work, whereas older age groups tend to engage in less physically intensive work^25^. The current study demonstrates that energy consumption was significantly low in workers aged 60 years or older, but HRI risk did not decrease in that age group. Physical work capacity of a 65 year old can be reduced by up to ∼50% compared with an average 25 year old^32^. Older worker might feel higher physical strain than young worker. That is because HRI risk in elderly increase even in light workloads.

Regarding construction experience, it was significantly associated with reduced HRI risk. We previously reported that experience of outdoor manual work reduced risk of HRI^24^. Lack of experience is also reported to increase HRI risk^33^, and younger people tend to pay little attention to preventive measures for HRI^31^. Education for workers with little experience is necessary to raise awareness of HRI.

In the present study, maximum skin temperature was significantly associated with HRI risk. Ideally, core body temperature should be used to monitor HRI risk. To assess body temperature from the skin, measurements should be taken from more than four different points of the body^34^. Skin temperature may not increase proportionally to rapidly increasing core body temperature. The ratio of core-skin temperature can vary between 0.9/0.1 (core/skin) on rectal temperature ≤ 36.8 °C and 0.7/0.3 on rectal temperature ≥ 39.0 °C for the vasodilated and vasoconstricted skin, respectively^21^. However, rapidly elevating skin temperature indicates elevating core body temperature so that skin temperature is an effective index for HRI risk evaluation.

Working at Site 2 had a significantly higher OR for HRI risk in the present study. WBGT recorded at Site 2 was higher than that at Site 1, but maximum WBGT did not show a significant relationship to HRI risk. And more, most of the personal data did not show significant difference and the hypertension was recorded only in Site 1. Some preventive measures, such as having a rest space, allocating rest time, and encouraging water intake, were taken in both sites. These measures were thought to be the reason why a significant relationship was not observed between WBGT and HRI risk. The American Conference of Governmental Industrial Hygienists (ACGIH) determined the WBGT threshold by workload^35^. As we could not get the data of workload, we used the energy consumption data instead, which however did not reflect the workload accurately. By clearing the differences in detailed occupational conditions such as work load and management between the two sites, more effective measures for HRI may be employed. Further investigation is required.

This study has several limitations. First, we could not obtain data regarding the workers’ clothing. HRI occurs as a result of complex factors, and clothing is strongly related to physiological performance^23^. Second, the wearable sensor used to obtain vital information in every 3 min in the current study had missing data due to mechanical reasons. We could not estimate the rate of change in skin temperature and its effect on heat strain.

In the present study, age, working area, heart rate immediately after warming up, maximum skin temperature and experience of construction were all significantly related to an increased heart rate indicative of HRI risk. Increased heart rate immediately after warming up may indicate morning fatigue due to, for example, insufficient sleep, drinking alcohol the night previously and sickness. Asking workers about their condition each morning may lead to effective preventive measures of HRI during work. For occupational risk management, monitoring environmental and personal conditions is essential. This study demonstrated the usefulness of continuous monitoring for HRI risk. Except for immediately after warming up, there might be pattern that predict HRI risk. We indicated a potential of continuous monitoring for the prevention of HRI. The importance of continuous monitoring should be increased in order to reduce the occurrence of occupational HRI.

## Methods

### Study design

This study was conducted as a cross-sectional study. We conducted an exploratory research about factors associated with heart rate of HRI risk among construction workers.

### Study subjects

Study subjects were construction workers, who were employed by a subcontractor, and a prime contractor directed all working procedures and managed the workers’ data. The prime contractor managed two sites where the workers were engaged in work during July 1st to September 30th, 2019, which was determined “a period to strengthen the prevention of occupational injury” in Japan. Prime contractor recruited to join this study and 84 (53 in Site 1 and 31 in Site 2) agreed to participate in the study. However, 23 were excluded due to monitoring failure and later refusal. Finally, we analysed vital data of 61 (35 in Site 1 and 26 in Site 2) workers. All subjects were male.

### Data collection

We obtained personal, vital, and environmental data of 61 workers from the prime contractor. The personal data were age, sex, past medical histories (hypertension and diabetes), duration of career in construction (years) and days worked at the site being studied.

The vital data were collected using a wearable sensor, LW-360HR (GISupply, Inc. Japan), worn on the left wrist of all workers. The sensor measured and recorded skin temperature of the ankle (°C), heart rate (BPM), and energy consumption (kcal) every three minutes, starting at the beginning of the working day immediately after warming up, and finishing at the end of the working day.

The environmental data were collected by using C-BB-15 cm (CLIMATEC, Inc. Japan) and HMP60 Humidity and Temperature Probe (Vaisala Corporation. Finland) at Site 1 and Davis Vantage Pro 2 (Keisoku Net Service Co., Ltd. Japan) at Site 2. The measurement method was based on ISO 7243: 1989^19^. WBGT was recorded every 10 min.

In the present study, heart rate with high-risk HRI was determined daily maximum heart rate using the following formula: 180 − 0.65 × age, based on the limit of heart rate in workplace determined by ISO 9886: 2004^34^. We determined high-risk group for the days scored high-risk HRI low-risk group was that heart rate did not archive to high-risk HRI.

### Statistical analysis

We used R 3.6.1 for all statistical analyses. All data are described as mean (SD: standard deviation), median (25–75 percentile), or n (%). Personal data and environmental data, and heart rate with HRI risk (high risk vs. low risk) were compared between the two sites. To investigate the relationship between workload and age, we divided the subjects into four age groups (< 40, 40–49, 50–59, and ≥ 60 years) and compared daily energy consumption using analysis of variance (ANOVA). A logistic regression model was conducted to determine the factors that were related to heart rate with high-risk HRI. Age (continuous variable), working days, experience of construction, hypertension, diabetes, working site, employer (subcontractor or sub-subcontractor), maximum skin temperature, energy consumption, heart rate immediately after warming up, maximum WBGT (< 25, 25–28, 28–31, > 31 °C) and WBGT difference in a day were selected as dependent variables. WBGT was categorized according to the guidelines for prevention of heat disorders for sporting activities^36^. The adjusted ORs for heart rate with high-risk HRI, and their 95% confidence intervals (95% CI), were calculated.

### Ethics

We confirmed that all research was performed in accordance with Ethical Guidelines for Medical and Health Research Involving Human Subjects^37^. This study was approved by the Ethics Committees of Fukushima Medical University (Application No. 2019–185). The prime contractor explained the study to their workers and obtained written informed consent for participation in the study and data collection. All data was anonymized by the prime contractor before being passed on to us.

## Data Availability

No additional data are available for this study. However, inquiries concerning the data may be made to the corresponding author.
